# Application of the ICD-11 classification of personality disorders

**DOI:** 10.1186/s12888-018-1908-3

**Published:** 2018-10-29

**Authors:** Bo Bach, Michael B First

**Affiliations:** 1 Center of Excellence on Personality Disorder, Psychiatric Research Unit, Region Zealand, Slagelse Psychiatric Hospital, Fælledvej 6, Bygning 3, 4200 Slagelse, Denmark; 2 Department of Psychiatry, New York State Psychiatric Institute, Columbia University, New York, NY USA

**Keywords:** ICD-11, Classification, Personality disorder, Severity, Trait

## Abstract

**Background:**

The ICD-11 classification of Personality Disorders focuses on core personality dysfunction, while allowing the practitioner to classify three levels of severity (Mild Personality Disorder, Moderate Personality Disorder, and Severe Personality Disorder) and the option of specifying one or more prominent trait domain qualifiers (Negative Affectivity, Detachment, Disinhibition, Dissociality, and Anankastia). Additionally, the practitioner is also allowed to specify a Borderline Pattern qualifier. This article presents how the ICD-11 Personality Disorder classification may be applied in clinical practice using five brief cases.

**Case presentation:**

(1) a 29-year-old woman with Severe Personality Disorder, Borderline Pattern, and prominent traits of Negative Affectivity, Disinhibition, and Dissociality; (2) a 36-year-old man with Mild Personality Disorder, and prominent traits of Negative Affectivity and Detachment; (3) a 26-year-old man with Severe Personality Disorder, and prominent traits of Dissociality, Disinhibition, and Detachment; (4) a 19-year-old woman with Personality Difficulty, and prominent traits of Negative Affectivity and Anankastia; (5) a 53-year-old man with Moderate Personality Disorder, and prominent traits of Anankastia and Dissociality.

**Conclusions:**

The ICD-11 Personality Disorder classification was applicable to five clinical cases, which were classified according to Personaity Disorder severity and trait domain qualifiers. We propose that the classification of severity may help inform clinical prognosis and intensity of treatment, whereas the coding of trait qualifiers may help inform the focus and style of treatment. Empirical investigation of such important aspects of clinical utility are warranted.

**Electronic supplementary material:**

The online version of this article (10.1186/s12888-018-1908-3) contains supplementary material, which is available to authorized users.

## Background

Personality Disorder is important to all health care practitioners because it is a prevalent condition that applies to approximately 12% of the general community [1], 25% of primary care patients [2], and at least 50% of psychiatric outpatients [3]. This potentially complicates the relationship between patients and health care professionals, increases the risk of premature mortality, and results in a huge cost to society [4]. However, research highlights significant problems with the ICD-10 and DSM-IV/DSM-5 categorical approaches to Personality Disorder diagnostics, including arbitrary diagnostic thresholds, extensive overlap among categories, lack of evidence for 10 distinct categories, and insufficient clinical utility [4–6]. In comparison to the assessment of other mental disorders, assessment of Personality Disorders is more difficult in routine clinical practice. Reviewing the 79 DSM criteria for 10 disorders (plus 15 criteria of conduct disorder) is cumbersome and requires specialized training. In response to these shortcomings, the 11th edition of the International Classification of Diseases (ICD-11) adopts a dimensional approach to the classification of Personality Disorders that focuses on global level of severety and five trait qualifiers [7]. The present article aims to introduce and illustrate how the ICD-11 Personality Disorder classification may be applied in clinical practice using five brief cases with different diagnostic features.

### Rationale of the ICD-11 classification of personality disorders

The ICD-11 nomenclature for Personality Disorders [8] focuses on the impairment of self and interpersonal personality functioning, which may be classified according to degree of severity (“Personality Difficulty”, “Mild Personality Disorder”, “Moderate Personality Disorder”, and “Severe Personality Disorder”). Furthermore, the diagnosis may also be specified with one or more prominent trait qualifiers (Negative Affectivity, Detachment, Dissociality, Disinhibition, and Anankastia), which contribute to the impairment in personality functioning. Unlike the polythetic ICD-10 criteria for Personality Disorders (e.g., five out of nine criteria) which set the disorder/non-disorder threshold based on the number of criteria that are met, the ICD-11 diagnostic requirements for Personality Disorders base the diagnosis on a global evaluation of personality functioning. Given that personality functioning might be impaired in various ways, the trait qualifiers are available to describe the specific pattern of traits that contribute to the global personality dysfunction. The general diagnostic requirements for Personality Disorder are presented in Table 1, the guidelines for determination of the level of severity are presented in Tables 2, 3 and 4 and the five trait domain qualifiers are elucidated in Table 5. In addition to specifying Personality Disorder severity and stylistic trait qualifiers, the user is also allowed to code substhreshold Personality Difficulty and a Borderline Pattern qualifier (see Table 6).

**Table 1:** General diagnostic requirements

● An enduring disturbance characterized by problems in functioning of aspects of the self (e.g., identity, self-worth, accuracy of self-view, self-direction), and/or interpersonal dysfunction (e.g., ability to develop and maintain close and mutually satisfying relationships, ability to understand others’ perspectives and to manage conflict in relationships). ● The disturbance has persisted over an extended period of time (> 2 years). ● The disturbance is manifest in patterns of cognition, emotional experience, emotional expression, and behaviour that are maladaptive (e.g., inflexible or poorly regulated). ● The disturbance is manifest across a range of personal and social situations (i.e., is not limited to specific relationships or social roles), though it may be consistently evoked by particular types of circumstances but not others. ● The patterns of behaviour characterizing the disturbance are not developmentally appropriate and cannot be explained primarily by social or cultural factors, including socio-political conflict. ● The symptoms are not due to the direct effects of a medication or substance, including withdrawal effects, and are not better explained by another Mental and Behavioural Disorder, a Disease of the Nervous System, or another health condition. ● The disturbance is associated with substantial distress or significant impairment in personal, family, social, educational, occupational or other important areas of functioning.	

*Note*. Adapted from the ICD-11 Clinical Descriptions and Diagnostic Guidelines for Personality Disorder.

**Table 2:** Aspects of personality functioning that contribute to severity determination in Personality Disorder

Degree and pervasiveness of disturbances in functioning of aspects of the self: ● Stability and coherence of one’s sense of identity (e.g., extent to which identity or sense of self is variable and inconsistent or overly rigid and fixed). ● Ability to maintain an overall positive and stable sense of self-worth. ● Accuracy of one’s view of one’s characteristics, strengths, limitations. ● Capacity for self-direction (ability to plan, choose, and implement appropriate goals).	
Degree and pervasiveness of interpersonal dysfunction across various contexts and relationships (e.g., romantic relationships, school/work, parent-child, family, friendships, peer contexts): ● Interest in engaging in relationships with others. ● Ability to understand and appreciate others’ perspectives. ● Ability to develop and maintain close and mutually satisfying relationships. ● Ability to manage conflict in relationships.	
Pervasiveness, severity, and chronicity of emotional, cognitive, and behavioral manifestations of the personality dysfunction: Emotional manifestations ○ Range and appropriateness of emotional experience and expression. ○ Tendency to be emotionally over- or underreactive. ○ Ability to recognize and acknowledge unwanted emotions (e.g., anger, sadness). Cognitive manifestations ○ Accuracy of situational and interpersonal appraisals, especially under stress. ○ Ability to make appropriate decisions in situations of uncertainty. ○ Appropriate stability and flexibility of belief systems. Behavioural manifestations ○ Flexibility in controlling impulses and modulating behaviour based on the situation and consideration of the consequences. ○ Appropriateness of behavioural responses to intense emotions and stressful circumstances (e.g., propensity to self-harm or violence).	
The extent to which the dysfunctions in the above areas are associated with distress or impairment in personal, family, social, educational, occupational or other important areas of functioning.	

*Note*. Adapted from the ICD-11 Clinical Descriptions and Diagnostic Guidelines for Personality Disorder

**Table 3:** Essential features of Personality Disorder severity

Mild Personality Disorder	Moderate Personality Disorder	Severe Personality Disorder
Disturbances affect some areas of personality functioning but not others (e.g., problems with self-direction in the absence of problems with stability and coherence of identity or self-worth; see Table 2), and may not be apparent in some contexts.	Disturbances affect multiple areas of personality functioning (e.g., identity or sense of self, ability to form intimate relationships, ability to control impulses and modulate behaviour; see Table 2). However, some areas of personality functioning may be relatively less affected.	There are severe disturbances in functioning of the self (e.g., sense of self may be so unstable that individuals report not having a sense of who they are or so rigid that they refuse to participate in any but an extremely narrow range of situations; self view may be characterized by self-contempt or be grandiose or highly eccentric; see Table 2).
There are problems in many interpersonal relationships and/or in performance of expected occupational and social roles, but some relationships are maintained and/or some roles carried out.	There are marked problems in most interpersonal relationships and the performance of most expected social and occupational roles are compromised to some degree. Relationships are likely to be characterized by conflict, avoidance, withdrawal, or extreme dependency (e.g., few friendships maintained, persistent conflict in work relationships and consequent occupational problems, romantic relationships characterized by serious disruption or inappropriate submissiveness).	Problems in interpersonal functioning seriously affect virtually all relationships and the ability and willingness to perform expected social and occupational roles is absent or severely compromised.
Specific manifestations of personality disturbances are generally of mild severity (see examples in Table 4).	Specific manifestations of personality disturbance are generally of moderate severity (see examples in Table 4).	Specific manifestations of personality disturbance are severe (see examples in Table 4) and affect most, if not all, areas of personality functioning.
Is typically not associated with substantial harm to self or others.	Is sometimes associated with harm to self or others.	Is often associated with harm to self or others.
May be associated with substantial distress or with impairment in personal, family, social, educational, occupational or other important areas of functioning that is either limited to circumscribed areas (e.g., romantic relationships; employment) or present in more areas but milder.	Is associated with marked impairment in personal, family, social, educational, occupational or other important areas of functioning, although functioning in circumscribed areas may be maintained.	Is associated with severe impairment in all or nearly all areas of life, including personal, family, social, educational, occupational, and other important areas of functioning.

*Note*. The diagnostic guideline should be accompanied with the examples provided in Table 4. Adapted from the ICD-11 Clinical Descriptions and Diagnostic Guidelines for Personality Disorder. All five levels of personality functioning are described and exemplified in Additional file 1

**Table 4:** Examples of specific disturbances in personality functioning

Mild Personality Disorder	Moderate Personality Disorder	Severe Personality Disorder
The individual’s sense of self may be somewhat contradictory and inconsistent with how others view them.	The individual’s sense of self may become incoherent in times of crisis.	The individual’s self-view is very unrealistic and typically is highly unstable or internally contradictory.
The individual has difficulty recovering from injuries to self-esteem.	The individual has considerable difficulty maintaining positive self-esteem or, alternatively, has an unrealistically positive self-view that is not modified by evidence to the contrary.	The individual has serious difficulty with regulation of self-esteem, emotional experience and expression, and impulses, as well as other aspects of behaviour (e.g., perseveration, indecision).
The individual’s ability to set appropriate goals and to work towards them is compromised; the individual has difficulty handling even minor setbacks.	The individual exhibits poor emotion regulation in the face of setbacks, often becoming highly upset and giving up easily. Alternatively, the individual may persist unreasonably in pursuit of goals that have no chance of success.	The individual is largely unable to set and pursue realistic goals.
The individual may have conflicts with supervisors and co-workers, but is generally able to sustain employment.	The individual may exhibit little genuine interest in or efforts toward sustained employment.	The individual is unwilling or unable to sustain regular work due to lack of interest or effort, poor performance (e.g., failure to complete assignments or perform expected roles, unreliability), interpersonal difficulties, or inappropriate behaviour (e.g., fits of temper, insubordination).
The individual’s limitations in the ability to understand and appreciate others’ perspectives create difficulties in developing close and mutually satisfying relationships.	Major limitations in the ability to understand and appreciate others’ perspectives hinder developing close and mutually satisfying relationships.	The individual’s interpersonal relationships, if any, lack mutuality; are shallow, extremely one-sided, unstable, and/or highly conflictual, often to the point of violence.
There may be estrangement in some relationships, but relationships are more commonly characterized by intermittent or frequent, minor conflicts that are not so severe that they cause serious and long-standing disruption. Alternatively, relationships may be characterized by dependence and avoidance of conflict by giving in to others, even at some cost to themselves.	Problems in those relationships that do exist are common and persistent; may involve frequent, serious, and volatile conflict; and typically are quite one-sided (e.g., very strongly dominant or highly submissive).	Family relationships are absent (despite having living relatives) or marred by significant conflict. The individual has extreme difficulty acknowledging unwanted emotions (e.g., does not recognize or acknowledge experiencing anger, sadness, or other emotion).
Under stress, there may be some distortions in the individual’s situational and interpersonal appraisals but reality testing remains intact.	Under stress there are marked distortions in the individual’s situational and interpersonal appraisals. There may be mild dissociative states or psychotic-like beliefs or perceptions (e.g., paranoid ideas).	Under stress, there are extreme distortions in the individual’s situational and interpersonal appraisals. There are often dissociative states or psychotic-like beliefs or perceptions (e.g., extreme paranoid reactions).

*Note*. The examples should be accompanied with the diagnostic guideline provided in Table 3. Adapted from the ICD-11 Clinical Descriptions and Diagnostic Guidelines for Personality Disorder. All five levels of personality functioning are described and exemplified in Additional file 1

**Table 5:** Trait domain qualifiers that contribute to the expression of personality dysfunction

Trait domain	Core definition	Specific features
Negative Affectivity	A tendency to experience a broad range of negative emotions with a frequency and intensity out of proportion to the situation.	Anxiety, anger, worry, fear, vulnerability, hostility, shame, depression, pessimism, guilt, low self-esteem, and mistrustfulness. For example, once upset, such individuals have difficulty regaining their composure and must rely on others or on leaving the situation to calm down.
Detachment	A tendency to maintain interpersonal distance (social detachment) and emotional distance (emotional detachment)	*Social detachment* including avoidance of social interactions, lack of friendships, and avoidance of intimacy. *Emotional detachment* including being reserved, aloofness, and limited emotional expression and experience. For example, such individuals seek out employment that does not involve interactions with others.
Dissociality	Disregard for the rights and feelings of others, encompassing both self-centeredness and lack of empathy.	*Self-centeredness* including entitlement, grandiosity, expectation of others’ admiration, and attention-seeking. *Lack of empathy* including being deceptive, manipulative, exploiting, ruthless, mean, callous, and physically aggressive, while sometimes taking pleasure in others’ suffering. For example, such individuals respond with anger or denigration of others when they are not granted admiration.
Disinhibition	A tendency to act rashly based on immediate external or internal stimuli (i.e., sensations, emotions, thoughts), without consideration of potential negative consequences.	Impulsivity, distractibility, irresponsibility, recklessness, and lack of planning. For example, such individuals may be engaged in reckless driving, dangerous sports, substance use, gambling, and unplanned sexual activity.
Anankastia	A narrow focus on one’s rigid standard of perfection and of right and wrong, and on controlling one’s own and others’ behaviour and controlling situations to ensure conformity to these standards.	*Perfectionism* including concern with rules, norms of right and wrong, details, hyper-scheduling, orderliness, and neatness. *Emotional and behavioral constraint* including rigid control over emotional expression, stubbornness, risk-avoidance, perseveration, and deliberativeness. For example, such individuals may stubbornly redo the work of others because it does not meet their standards.

*Note*. Adapted from the ICD-11 Clinical Descriptions and Diagnostic Guidelines for Personality Disorder, which include a more detailed description of the trait domain qualifiers

**Table 6:** Borderline pattern qualifier

The Borderline pattern qualifier may be applied to individuals whose pattern of personality disturbance is characterized by a pervasive pattern of instability of interpersonal relationships, self-image, and affects, and marked impulsivity, as indicated by five (or more) of the following: ● Frantic efforts to avoid real or imagined abandonment. ● A pattern of unstable and intense interpersonal relationships, typically characterized by alternating between extremes of idealization and devaluation. ● Identity disturbance, manifested in markedly and persistently unstable self-image or sense of self. ● Impulsivity manifested in potentially self-damaging behaviours (e.g., risky sexual behaviour, reckless driving, excessive alcohol or substance use, binge eating). ● Recurrent episodes of self-harm (e.g., suicide attempts or gestures, self-mutilation). ● Emotional instability due to marked reactivity of mood. Fluctuations of mood may be triggered either internally (e.g., by one’s own thoughts) or by external events. As a consequence, the individual experiences intense dysphoric mood states, which typically last for a few hours but may last for up to several days. ● Chronic feelings of emptiness. ● Inappropriate intense anger or difficulty controlling anger manifested in frequent displays of temper (e.g., yelling or screaming, throwing or breaking things, getting into physical fights). ● Transient dissociative symptoms or psychotic-like features (e.g., brief hallucinations, paranoia) in situations of high affective arousal. Other manifestations of Borderline pattern, not all of which may be present in a given individual at a given time, include the following: ● A view of the self as inadequate, bad, guilty, disgusting, and contemptible. ● An experience of the self as profoundly different and isolated from other people; a painful sense of alienation and pervasive loneliness. ● Proneness to rejection hypersensitivity; problems in establishing and maintaining consistent and appropriate levels of trust in interpersonal relationships; frequent misinterpretation of social signals.	

*Note*. Adapted from the ICD-11 Clinical Descriptions and Diagnostic Guidelines for Personality Disorder

As shown in Table 2 and 3, the classification of severity aligns with the psychodynamic tradition of personality organization [9, 10] as well as scientifically valid models of core Personality Disorder features [11–15]. Importantly, research shows that much of the predictive and prognostic value in Personality Disorder assessment can be derived from such a core dimension [13, 16]. A classification according to severity also provides information for guiding intensity of clinical management and treatment [10, 17, 18].

Finally, as shown in Table 5, the deliniation of five trait domain qualifiers aligns with other empirically-derived dimensional schemes, including the cross-culturally replicated Five-Factor Model [19–21] and the DSM-5 Alternative Model of Personality Disorders [22–24]. The ICD-11 trait domain qualifiers not only provide scientifically sound and homogenous building blocks of personality psychopathology but also clinical information for selecting type and focus of treatment [25–28].

## Application of the ICD-11 model in clinical practice

At a basic level, the ICD-11 classification allows the clinician the option of rapid assessment of personality functioning. As such, a practitioner should be able first to identify the presence or absence of Personality Disorder, then its severity, and, if appropriate, one or more prominent trait qualifiers that contribute to the expression of personality dysfunction. Accordingly, the procedure for classification of ICD-11 Personality Disorder is fairly similar to the procedure of diagnosing ICD-10 F32 Depressive episode which has three levels of severity (*mild*, *moderate*, and *severe*), and which may, if appropriate, be further qualified by additional codes for individual features. For example, F32.11 Moderate depressive episode with somatic syndrome or F32.3 Severe depressive episode with psychotic symptoms.

### Classification of personality disorder severity replaces comorbidity

Because the ten different types of categorical Personality Disorder diagnoses no longer exist in the ICD-11 classification, the practitioner has no choice but to assess Personality Disorder itself rather than focussing the assessment on overlapping and heterogenous polythetic categories (see Tables 1 and 2) [4]. Accordingly, instead of the classification into ten types, the ICD-11 can be said to involve a subclassification into three categories of severity, which cannot co-exist with one another (i.e., a patient cannot have a Mild Personality Disorder while also having a Severe Personality Disorder). Thus, the ICD-11 classification eradicates the excessive comorbidity characterizing the different ICD-10 Personality Disorder categories. However, the clinician still has the option of indicating the presence of a Personality Disorder without specifying its severity (i.e., “severity unspecified”). The specified severity threshold for yielding a Personality Disorder diagnosis (at least “mild” severity) is explained in Table 3 and exemplied in Table 4. Thus, the definition of “mild” severity may also be employed as a screener for presence or absence of Personality Disorder.

### The option of coding subthreshold personality difficulty

In addition to the Personality Disorder diagnosis (in the chapter on Mental and behavioral disorders), clinicians have the option of indicating the presence of Personality Difficulty. Personality Difficulty is not considered to be a mental disorder per se, but is availble for clinical use and is located in the section of the ICD-11 classification for non-disease entities that constitute factors influencing health status and encounters with health services. Personality Difficulty is somewhat akin to the ICD-10 non-disorder category Z73.1 “accentuation of personality traits” which is a subcategory of the Z73 “Problems Related to Life-Management Difficulty” in the chapter “Factors Influencing Health Status and Contact with Health Services”.

Like a Personality Disorder diagnosis, Personality Difficulty is characterized by relatively stable difficulties (e.g., at least 2 years). Such difficulties are associated with some problems in functioning which are insufficiently severe to cause notable disruption in social, occupational, and interpersonal relationships and that may be limited to specific relationships or situations. Problems with emotions, cognitions, and behaviors are only expressed intermittently (e.g., during times of stress) or at low intensity. In contrast to Mild Personality Disorder, the individual with Personality Difficulty only has some intermittent or low intensity personality-related problems (e.g., in circumscribed risk situations), but not to the extent that it compromises the individual’s ability to keep a job, initiate and maintain friendships, and have somewhat satisfactory intimate relationships.

For example, a patient with eating disorder may have personality difficulities of rigid perfectionism (i.e., Anankastia) while maintaining a strong social network and making slow but steady progress towards finishing an education. Another patient with resistant anxiety symptoms may have difficulties of anxiousness (i.e., Negative Affectivity) but otherwise be viewed as a treasured friend and collegue. In both cases, the specified patterns of Personality Difficulty reveal specific vulnerabilities. Taken together, when most appropriate a code of Personality Difficulty may be applied to the patient with noteworthy but not prominent personality problems.

### Personality trait qualifiers

One or more stylistic trait qualifiers may be coded if they are prominent in the personality makeup of the individual diagnosed with Personality Disorder or Personality Difficulty. Yet, it is important to recognize that the trait qualifiers are not like categories or syndromal diagnoses, but instead denote stylistic dimensions that contribute to the expression of the personality dysfunction. However, for the purpose of coding, the prominent trait qualifiers can only be indicated as present or absent even though they exist on a continuum. Essentially, the overall severity of personality dysfunction (i.e., *mild*, *moderate*, and *severe*) reflects the degree to which the prominent traits have an impact on the patient’s self- and interpersonal functioning [29], which is illustrated in a figure for each of the five cases. Thus, Severe Personality Disorder is likely to be associated with several trait domain qualifiers, whereas Mild Personality Disorder may be associated with the presence of only one trait qualifier. In other words, complexity of trait domain qualifiers may often reflect the severity of the Personality Disorder. However, in some cases an individual may have a Severe Personality Disorder and manifest only one prominent trait qualifier (e.g., Dissociality causing severe danger towards others).

### Borderline pattern qualifier

As presented in Table 6, the ICD-11 classification of Personality Disorders also includes the option of specifying a *Borderline Pattern Qualifier*. Like the trait qualifiers, the Borderline Pattern qualifier is considered optional and can be used in combination with the trait qualifiers (e.g., *Moderate Personality Disorder, with Borderline Pattern, with Negative Affectiviy, Disinhibition, and Dissociality*). Unlike the trait qualifiers, the Borderline Pattern Qualifier is operationalized as requiring at least 5 out of 9 polythetic features adapted from the DSM-5 criteria for Borderline Personality Disorder. It has been suggested that this qualifier may serve as a familiar indicator for choosing psychotherapeutic treatment consistent with established theory and treatment manuals.

### Onset and stability of personality disorder

As presented in Table 1, the personality disturbance must have persisted over an extended period of time (> 2 years). Elements of Personality Disorder tend to first appear in childhood or adolescence and continue to be manifest into adulthood. However, while ICD-10 states that Personality Disorders tend to be stable over time, the ICD-11 guideline explicitly states that Personality Disorders are only “relatively” stable after young adulthood, and may change such that a person who had a Personality Disorder during young adulthood no longer has one by middle age. In some cases, a person who earlier did not have a diagnosable Personality Disorder, may develop one later in life. Sometimes, emergence of Personality Disorder in older adults may be related to the loss of social supports that had previously helped to compensate for personality disturbance.

### Features of psychoticism and level of severity

In contrast to the DSM-5 Section II and Section III approaches, the ICD-11 classification does not provide any code for Schizotypal Personality Disorder or Psychoticism because such features are coded within Schizophrenia and other primary psychotic disorders. However, as shown in Tables 3 and 4, the ICD-11 classification of Personality Disorder severity may be based on whether the patient experiences “dissociative states or psychotic-like beliefs or perceptions” and/or is “highly eccentric”, which may resemble certain features of Schizotypal Personality Disorder. This is consistent with the traditional structural approach to classification of personality organization (e.g., high, middle, and low borderline levels) [9, 10], in which the lowest and most severe level may involve transient psychotic states. In other words, the ICD-11 approach classifies the capacity for reality testing (i.e., accuracy of situational and interpersonal appraisals) according to level of Personality Disorder severity and not as a distinct type or trait domain. However, as shown in Table 6, the *Borderline Pattern qualifier* also involves “Transient dissociative symptoms or psychotic-like features (e.g., brief hallucinations, paranoia) in situations of high affective arousal,” which is consistent with the established DSM-IV/5 construct of Borderline Personality Disorder*.*

### How to operationalize the ICD-11 personality disorder diagnosis?

After having ensured that the general diagnostic requirements for Personality Disorder are met (Table 1), the user may select one of three different diagnostic codes according to Personality Disorder severity (Table 3), followed by the option of coding one or more prominent trait qualifiers (Table 5). Additionally, the Borderline Pattern qualifier may also be applied if the clinical description matches this pattern (Table 6). As in the ICD-10, the relevant information may be gathered from clinical interviews and observations, review of clinical records, and/or informant reports.

Assessment tools are curently being developed to assist clinicians and researchers in the assessment of Personality Disorder diagnosis according to ICD-11. In the meantime, diagnostic information obtained from assessment tools developed for the DSM-5 AMPD model can be used for making an ICD-11 dimensional Personality Disorder diagnosis. For example, the Structured Clinical Interview for the DSM-5 Alternative Model of Personality Disorders (SCID-AMPD) operationalizes personality functioning according to the DSM-5 Level of Personality Functioning Scale (LPFS) along with the 25 DSM-5 trait facets [30]. The LPFS score along with the 25-facet personality profile can be converted into an ICD-11 Personality Disorder diagnosis using a “cross walk” as described in Table 7. Accordingly, SCID-AMPD Module I evaluates three levels of Personality Disorder impairment (the two lower levels comprise subthreshold for diagnosis and healthy functioning, respectively) [31], which translate into the ICD-11 classification of Mild Personality Disorder, Moderate Personality Disorder, and Severe Personality Disorder as illustrated in Table 7. Likewise, the SCID-AMPD Module II evaluates DSM-5 trait facets and domains [30], which may be translated into ICD-11 trait domain qualifiers directly (see Table 7) or deliniated by means of an algorithm for trait facets measured with the Personality Inventory for DSM-5 (PID-5)^1^ [32]. Finally, the ICD-11 trait domain qualifiers may also be derived from available ICD-10 categorical Personality Disorder information using the “cross walk” presented in Table 8 [22].

**Table 7:** ICD-11 “Cross Walk” for DSM-5 Alternative Model of Personality Disorders

ICD-11 Severity of Personality Dysfunction	DSM-5 Criterion A: Level of Personality Functioning
None	0) No impairment (Healthy Functioning)
Personality Difficulty	1) Some impairment
Mild Personality Disorder	2) Moderate impairment
Moderate Personality Disorder	3) Severe impairment
Severe Personality Disorder	4) Extreme impairment
ICD-11 Trait Domain Qualifiers	DSM-5 Criterion B: Trait Domains
Negative Affectivity	Negative Affectivity
Detachment	Detachment
Disinhibition	Disinhibition
Dissociality	Antagonism
Anankastia	[Rigid Perfectionism and Perseveration]^a^

*Note*. The threshold for a Personality Disorder diagnosis is a t least Mild Personality Disorder (ICD-11) or Moderate impairment of personality functioning (DSM-5)

^a^These are facets from the domains of (low) Disinhibition and (high) Negative Affectivity, respectively

**Table 8:** Tentative ICD-10 “Cross Walk” for ICD-11 Trait Domain Qualifiers

ICD-10 Category	ICD-11 Qualifier	Specific ICD-11 Trait Features
*F60.0 Paranoid*	Negative Affectivity	Mistrustfulness, anger, bitterness, tendency to hold grudges; may become overwrought over real or perceived slights or insults from others.
	Detachment	Emotional and interpersonal distance; avoidance of close friendships.
*F60.1 Schizoid*	Detachment	Do not enjoy intimacy or social interactions and are not particularly interested in sexual relations; aloofness, emotional unexpressiveness, non-reactive to negative and positive events, with a limited capacity for enjoyment.
	low Negative Affectivity	Absence of emotional intensity and sensitivity.
*F60.2 Dissocial*	Dissociality	Lack of empathy including callous, deceptive, manipulative, exploiting, mean, ruthless, and physically aggressive behavior, and may sometimes take pleasure in inflicting pain or harm.
	Disinhibition	Impulsivity, irresponsibility, recklessness, and lack of planning without regard for risks or consequences.
	low Negative Affectivity	Absence of vulnerability, shame, and anxiety.
*F60.3 Emotionally unstable*	Negative Affectivity	Poor emotion regulation including being overreactive to criticism, problems, and setbacks; low frustration tolerance; often experiencing and displaying multiple emotions simultaneously or vacillate among a range of emotions in a short period of time. Once upset, it is difficult to regain composure.
	Disinhibition	Impulsivity associated with e.g., substance use, unplanned sexual activity, and sometimes deliberate self-harm; lack of planning.
	Dissociality	Sometimes being mean and physically aggressive.
*F60.4 Histrionic*	Dissociality	Expectation of others’ admiration and attention-seeking behaviours to ensure being the center of others’ focus.
	Disinhibition	Easily distracted by extraneous stimuli, such as others’ conversations and tend to scan the environment for more enjoyable options. Acts rashly based on whatever is attractive at the moment. Focus on immediate feelings and sensations.
	Negative Affectivity	Emotional lability including being overreactive to external events; often experiences and displays multiple emotions simultaneously.
	low Detachment	Reversed emotional and social detachment including avoidance of social interactions, limited emotional expression and experience.
*F60.5 Anankastic*	Anankastia	Perfectionism including hyper-scheduling, planfulness, orderliness, and neatness. Behavioral constraint including control over emotional expression, stubbornness, risk-avoidance, perseveration, and deliberativeness.
	low Disinhibition	Reversed irresponsibility, lack of Planning, and impulsivity.
	Negative Affectivity	Worry, anxiety, and negativistic attitudes involving rejection of other’s suggestions or advice.
*F60.6 Anxious (avoidant)*	Negative Affectivity	Anxiety, vulnerability, fear, shame, and low self-esteem/confidence including avoidance of situations and activities that are judged too difficult.
	Detachment	Avoidance of social interactions and intimacy, seek out employment that does not involve interactions with others, and even refuse promotions if it would entail more interaction with others.
	low Dissociality	Reversed self-centeredness: attention-seeking behaviours to ensure being the center of others’ focus; believing that one has have many admirable qualities, that one’s accomplishments are outstanding, that one will achieve greatness, and that others should admire one.
*F60.7 Dependent*	Negative Affectivity	Anxiety, vulnerability, and low self-confidence including *dependency*, which may be manifested in frequent reliance on others for advice, direction, and other kinds of help.
	low Dissociality	Excessive prosocial behavior and absence of self-centeredness: lack of concern about own needs, desires, and comfort, while those of others are overly considered.
*F60.8 Other: Narcissistic*	Dissociality	Grandiosity, a sense of entitlement, believing that they have many admirable qualities, that they have or will achieve greatness, and that others should admire them.
	Negative affectivity	Dysregulated self-esteem, which may involve envy of others’ abilities and indicators of success; the individual can become overwrought over real or perceived slights or insults.

For clinical screening and research purposes, self-report measures have been developed to deliniate severity of personality dysfunction and prominent trait qualifiers. For example, the Level of Personality Functioning Scale – Brief Form 2.0 (LPFS-BF) [33, 34] efficiently measures impairment of self- and interpersonal functioning consistent with the ICD-11 diagnostic guidelines. The Personality Inventory for ICD-11 (PiCD) is a 60-item self-report or informant-report instrument, which describes the five ICD-11 domains [19]. Finally, as previously asserted, the ICD-11 domains may also be deliniated using an empirically established algorithm for using the ratings on the Personality Inventory for DSM-5 (PID-5) to determine the ICD-11 trait domain qualifiers [32].

## Case presentation

The following five cases demonstrate how the ICD-11 Personality Disorder classification may be applied to individuals with varying severity of personality dysfunction and configuations of trait qualifiers. All five cases meet the general diagnostic requirements for Personality Disorder, except for Case 4 (Fig. 4) whose clinical presentation is only characterized by subthreshold Personality Difficulty.

**Fig. 1:**
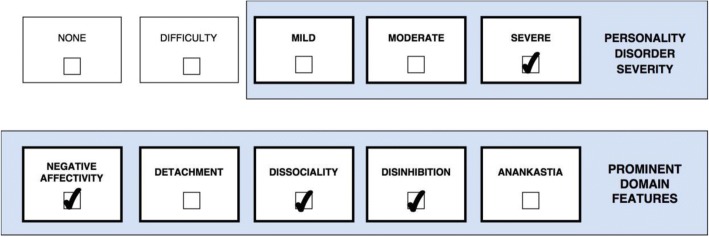
Severe Personality Disorder with Borderline Pattern and prominent traits of Negative Affectivity, Dissociality, and Disinhibition

Case 1 (Fig. 1) is a 29-year-old women, who has a history of numerous serious suicide attempts resulting in repeated hospitalizations, multiple treatment providers, and medication trials typically with little to no benefit. She has been diagnosed with ICD-10 F60.3 Emotionally unstable Personality Disorder, but her clinical presentation is also complicated by substance abuse (i.e., cannabis and amphetamine), eating disorder, panic attacks, aggressive/impulsive behaviors leading to a total loss of reliable friends, and severe self-harm that has endangered her life. During her childhood she was verbally and physically abused by her mother, and sexually abused by two of her mother’s male acquantances; she never knew her dad. Under stress she suffers from trauma-related dissociative states including symptoms of depersonalization and psychotic-like voices telling her to punish herself or vanish from the present reality, though, she is mostly aware that the voices only exist in her mind. When experiencing minor defeats or perceived rejection, she responds with feelings of self-loathing or anger. Due to excessive mistrust of other people, her ability to form intimate relationships and capacity for empathy is severily compromised, and she has no idea what to do with her life or what she has to offer. Apart from experiencing mistrust, emptiness, and anger, she occasionally uses ingratiation and charm in her attempts to have her need for warmth and approval met. As displayed in the figure, Case 1’s (Fig. 1) clinical presentation is classified as *Severe Personality Disorder* (e.g., serious difficulty with regulation of emotional experience, self-esteem, and impulses with a past history and future expectation of severe harm to self, psychotic-like perceptions, and she lacks reliable friends) with prominent trait qualifiers of *Negative Affectivity* (e.g., experiences negative emotions that are out of proportion to the situation including shame, mistrustfulness, and anger), *Disinhibition* (e.g., tendency to act impulsively in response to immediate stimuli in a harmful manner), and *Dissociality* (e.g., mistrust-related aggression and tendency to manipulate or seduce others). In this case *Moderate Personality Disorder* does not apply because Case 1 (Fig. 1) is not even able to maintain a few friendships or a regular job, and her self-injuries have caused long-term damage and endangered her life. Additionally, Case 1’s (Fig. 1) diagnosis may be further elucidated using the Borderline Pattern qualifier as indicated by nearly all of the features presented in Table 6.

**Fig. 2:**
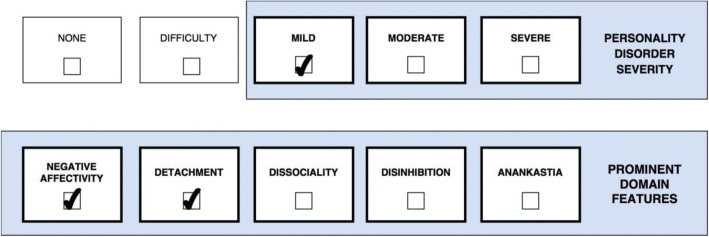
Mild Personality Disorder with prominent traits of Negative Affectivity and Detachment

Case 2 (Fig. 2) is a 36-year-old man with a history of panic attacks and recurrent depressive episodes. He is intelligent and sensitive but has not managed to finish any degree after high school. A psychiatric evaluation at an outpatient psychotherapy unit concluded that his personality features met ICD-10 criteria for F60.6 Avoidant Personality Disorder and F60.7 Dependent Personality Disorder. Case 2 (Fig. 2) grew up in a home with poor resources and a family climate characterized by emotional and physical neglect along with some emotional abuse by both parents. During adolescence, he suffered from loneliness, insecurity, poor self-worth, and self-defeating behaviors such as letting peers take advantage of him. He virtually had no friends in school and he generally felt anxious, shy, and unaccepted among peers. Accordingly, he was prone to act as an underdog or people-pleaser. These features were preserved in adulthood in terms of social withdrawal and intimacy avoidance in order not to feel criticized, ashamed, or rejected. However, today he maintains a permanent job and a couple of relationships beyond his two brothers. As displayed in the figure, Case 2’s (Fig. 2) clinical presentation is classified as *Mild Personality Disorder* (e.g., some distortions in interpersonal appraisal, difficulty maintaining positive self-esteem, is highly submissive in relationships but at least some healthy relationships and occupational roles are maintained) with prominent features of *Negative Affectivity* (e.g., anxiety, shame, low self-esteem, vulnerability, and depression depressivity) and *Detachment* (e.g., avoidance of social interactions). Notably, when Case 2 (Fig. 2) was younger, he would probably have been classified as *Moderate Personality Disorder* because he virtually had no friends; but he has improved since then as he now maintains a stable job and at least a couple of relationships.

**Fig. 3:**
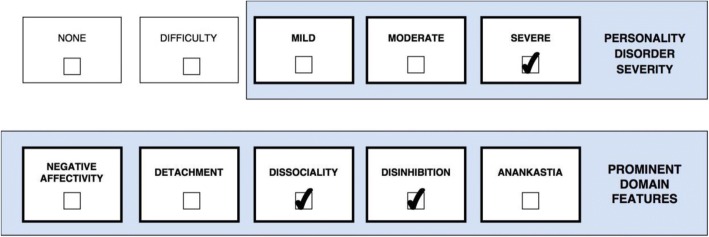
Severe Personality Disorder with prominent traits of Dissociality and Disinhibition

Case 3 (Fig. 3) is a 26-year-old man incarcerated for brutal violence (e.g., purposely injured a shop owner with a blunt instrument just to get his money). Although he claimed to feel no suffering from any symptoms or dysfunction, he sought rehabilitation for his dependency on cocaine which had caused him certain problems while imprisoned including withdrawal symptoms and symptoms of intoxication (e.g., tremor and dry mouth). A psychiatric evaluation concluded that his personality features met ICD-10 criteria for F60.2 Dissocial Personality Disorder including some characteristic psychopathic (e.g., callousness and exploitativeness) and narcissistic (e.g., entitlement) features as well as recklessness without concern for others’ safety. Case 3 (Fig. 3) did not recall much from his childhood and appeared aloof and emotionally detached while mentioning that his father was extremely physically abusive towards him and his mother. He did not experience anything positive from friendships, unless they could provide him with certain favors. Moreover, he was not ashamed of admitting that he did not care about harming others, but was rather proud of it, and he generally never felt any emotional or physical pain nor remorse. Case 3’s (Fig. 3) clinical presentation is classified as *Severe Personality Disorder* (e.g., past history and future expectation of severe harm to others, friendships have no genuine value to him, and self-view is characterized by entitlement) with prominent features of *Dissociality* (e.g., callousness, exploitation of others, and entitlement), *Disinhibition* (e.g., recklessness with no regard for others’ safety), and some *Detachment* (e.g., aloofness). In this case *Moderate Personality Disorder* would not apply because Case 3 (Fig. 3) is not even interested in maintaining a single friendship and the risk of dangerous harm to others is not just “sometimes” but “often” taking place.

**Fig. 4:**
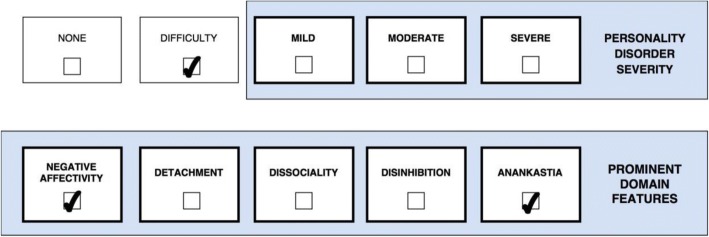
Personality Difficulity with prominent traits of Negative Affectivity and Anankastia

Case 4 (Fig. 4) is a 19-year-old highschool student, who was referred for treatment of ICD-10 F41.2 mixed anxiety and depressive disorder along with symptoms of anorexia nervosa, which she had previously been treated for in a private adolescent psychiatric clinic. Case 4 (Fig. 4) is from a relatively stable familiy, where the father works as physician and the mother as dentist. She has always been good at school and at finishing her duties in the home. Even though her parents have been busy with their own careers, they have persistently encouraged her to play the piano at different occasions and excel at horse riding competitions because they knew and expected that she was good at that. For that reason, her father never responded positively when she performed very well, whereas he showed disaoppointment if she did not get an A at her exams. While she was 13 her world fell apart as she discovered her father having an affair with another woman from his workplace, and she started overperforming in school and in sport while gradually developing eating disorder symptoms (restricting food leading to abnormally low weight) and even more unrelenting standards. However, she managed to maintain satisfying relationships with her friends as well as her mother and siblings. Case 4’s (Fig. 4) clinical presentation is primarily classified as *Anorexia Nervosa* in the context of *Personality Difficulty* (i.e., some long-standing difficulties in her way of thinking about the self and the world, including unrelenting standards, which are insufficiently severe to cause notable disruption in school and most relationships) with prominent features of *Negative Affectivity* (e.g., depressivity, shame, and anxiety) and *Anankastia* (e.g., perfectionism, concern with meeting obligations, perseveration, deliberatetiveness, and tight control of own emotional expression). In this case *Mild Personality Disorder* would not apply because Case 4’s (Fig. 4) habitual personality issues are not leading to any notable psychosocial impairment, whereas her problems are mainly attributable to other current mental problems.

**Fig. 5:**
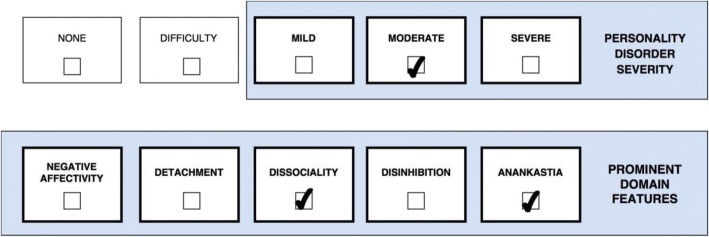
Moderate Personality Disorder with promiment traits of Anankastia and Dissociality

Case 5 (Fig. 5) is a 53-year-old highly skilled and well-groomed accountant who has worked for several companies during his carreer. At his current job, Case 5 (Fig. 5) was referred to a psychologist at the company’s HR department. Overall his personality characteristics were consistent with the ICD-10 Personality Disorder diagnoses F60.5 Anankastic Personality Disorder and F60.8 Other: Narcissistic Personality Disorder. Since adolescence, Case 5 (Fig. 5) has been more or less preoccupied with order, details, rules, and organization, including excessive pedantry and stubbornness. He always knew the “right” solution to most problems, and felt more capable of solving complicated things than nearly anyone else. Furthermore, he felt more important and entitled than most other people, and turned hostile when this was not recognized by others. Therefore, at work he has been reluctant to collaborate with others or to delegate “important” tasks to others, unless they submit to exactly his way of doing it. Colleagues and other people who know him well describes him as officious, supercilious, high-handed, unimaginative, intrusive, petty-minded, meddlesome, and nosy. An ex-wife has called Case 5 (Fig. 5) “a narcissist”, whereas he refers to her as “too vulnerable and unintelligent”. For those reasons he has not been able to maintain his occupational positions due to conflicts with superiors and emotional abuse of co-workers who he perceives as less efficient than himself. According to his account of things, it was his decision to leave the different companies during his career simply because they were not professional enough. According to ICD-11 guidelines, Case 5’s (Fig. 5) clinical presentation may be classified as *Moderate Personality Disorder* (e.g., a compromized capacity for understanding and appreciating others’ perspectives, work relationships are disrupted, and persistent conflicts result in emotional harm to others) with prominent traits of *Anankastia* (e.g., stubbornness, orderliness, and perfectionism) and *Dissociality* (e.g., entitlement, grandiosity, lack of empathy, meanness, and hostility). In this case, *Severe Personality Disorder* does not apply because while Case 5’s (Fig. 5) intimate and occupational relationships have been disrupted by frequent conflicts, he is still able to maintain work productivity and at least some relationships for a certain period of time. Likewise, *Mild Personality Disorder* does not apply because he is virtually incapable of or unwilling to sustain employment due to interpersonal issues, and he does not have any positive/healthy relationships not even with family members.

## Discussion

The ICD-11 approach changes the structure and process of diagnosing a Personality Disorder by focusing attention on the universal features of personality dysfunction including a classification of severity. Such a classification system has the advantage of simplifying the process of identifying a Personality Disorder. For example, the practitioner does not have to rule out the presence of a Personality Disorder by determining that none of the nine ICD-10 categories are present. Moreover, the ICD-11 classification is likely to have greater clinical utility because placing severity of personality functioning at the center of the diagnostic process can help service providers to distinguish those patients who have the greatest level of disturbance from those who do not, and thereby help services to target their interventions more effectively [13, 18].

The parsimoniousness of the ICD-11 classification may also frustrate some clinicians who desire a more detailed conceptualization of the patient’s personality stucture. Accordingly, in comparison to the nine categories in ICD-10 or the 25 facets in the DSM-5 AMPD model, the ICD-11’s five trait domains may be viewed as insufficiently detailed for describing all the subtle nuances of the patient’s personality. Yet, when all trait qualifier combinations are taken into account, the number of diagnostic constellations provides information for a more detailed clinical conceptualization. For example, when describing features of the ICD-10 diagnosis *Avoidant Personality Disorder*, the clinician may use the ICD-11 code *Mild Personality Disorder with prominent features of Negative Affectivity* (i.e., anxiousness and poor self-esteem) *and Detachment* (i.e., social withdrawal and intimacy avoidance). However, the ICD-11 classification does not accommodate a coding of the specific subfeatures or facets as in the DSM-5 AMPD model where *Avoidant Personality Disorder* may be described in terms of *anxiousness*, *withdrawal*, and *intimacy avoidance*. In any case, preliminary research suggests that the five ICD-11 trait domains explain a substantial amount of variance in all Personality Disorder categories [22, 35–38]. From this empirical perspective, little information (i.e. variance) seems lost in the transition from the 10 familiar Personality Disorder types to 5 trait domain qualifiers. However, this empirical reality may not necessarily be noticed by practitioners when using this new approach for the first time, and communication remains a central purpose of a diagnostic system.

The ICD-11 classification allows the clinician to apply as many trait domain qualifiers as necessary to portray the clinical reality and dynamics of personality functioning, which offers more unique diagnostic profiles in various combinations. For example, the trait domain qualifier of *Negative Affectivity* applies to both Case 1 and Case 2 (Figs 1 and 2) but with a substantially distinct “flavour” in each case due to the influence of co-occuring trait qualifiers. This is acknowledged in the ICD-11 guidelines, which indicates that the manifestation of one prominent trait is largely dependent on the presence of other traits. Accordingly, Case 1 (Fig. 1) is likely to experience “externalizing” features of *Negative Affectivity* (e.g., anger and contempt) due to her co-occuring Dissociality, whereas Case 2 (Fig. 2) is likely to experience “internalizing” features of *Negative Affectivity* (e.g., depression, anxiety, guilt) due to his co-occuring Detachment.

### Application of severity and traits in clinical treatment

In contrast to the ICD-10 Personality Disorder categories, the ICD-11 classification separates common features of personality dysfunction (e.g, capacity for self-direction and ability to understand others’ perspectives) from specific features of personality traits (e.g., impulsivity and attention seeking). This is consistent with research suggesting that severity of Personality Disorder tends to change or fluctuate while personality trait patterns tend to be relatively stable [6, 13, 16, 17]. From a treatment-perspective, traits tend to be resistant to change, whereas the severity of impairment related to the trait is less resistant to change. In other words, patients (and people in general) tend to stay essentially who they are, even if successful treatment helps them adapt who they are to their environment more effectively. Therefore, treatment should target what the Personality Disorder *does* to the patient (i.e., severity), as we cannot really change what it is (i.e., traits). For example, we estimated that Case 2’s (Fig. 2) disorder previously would have been classified as *Moderate Personality Disorder with prominent features of Negative Affectivity and Detachment* because he virtually had no friends beforehand. Due to his improvement he now maintains a stable job and at least a couple of relationships, and for that reason his disorder is now only classified as *Mild Personality Disorder* but still with the pattern of *Negative Affectivity* and *Detachment*. Likewise, an urgent goal of treating Case 1 (Fig. 1) would involve helping her regulate emotions in a less destructive manner so that her diagnosis may be changed from *Severe Personality Disorder* to *Moderate Personality Disorder* without getting rid of her basic style of *Negative Affectivity*, *Dissociality*, and *Disinhibition*. Similarly, a major goal of treating Case 3 (Fig. 3) would involve providing him with skills that may prevent him from being dangerous to others, and thereby changing his level of impairment from *Severe Personality Disorder* to *Moderate Personality Disorder*, while his dissocial core traits basically remain the same.

*Level of severity* tells the clinician important information about level of risk, prognosis, and treatment intensity, and it provides a variable for the assessment of change common to all individuals with a Personality Disorder diagnosis [13, 17]. Accordingly, the more severe a patient’s personality pathology, the greater the risk there is for extreme or problematic behavior (e.g., harm to self or others, treatment dropout, criminal issues, and psychotic-like symptoms) and the less optimistic the clinician can be for a smooth treatment with rapid and enduring gains [17]. Individuals with *Severe Personality Disorder* may need more intense treatments, such as hospitalization or multimodal approaches (e.g., combined group and individual). For example, Case 1 (Fig. 1) (*Severe Personality Disorder with prominent features of Negative Affectivity, Disinhibition, and Dissociality; Borderline Pattern qualifier*) would need a more intensive treatment program than Case 2 (Fig. 2) (*Mild Personality Disorder with prominent features of Negative Affectivity and Detachment*).

*Trait domain qualifiers* can be said to contribute to the more individual expression of personality dysfunction. For example, one patient may show impairment of the capacity for interpersonal functioning. Yet, it makes a great difference whether this impairment is related to being very dominant (e.g., *Dissociality*) or being overly submissive (e.g., *Negative Affectivity and Detachment*). Those two different trait expressions inform different treament foci and style. Moreover, knowing the patient’s prominent traits is useful for establishing a favorable treatment alliance, providing psychoeducation, increasing the patient’s self-knowledge, planning realistic treatment goals, and matching therapy to the patient’s personality (e.g., group therapy or individual therapy) [26]. Importantly, traits may also mirror habitual defensive- or coping responses (e.g., Detachment as a defense against shame or fears of being hurt by others) [28, 39, 40]. Therefore, traits may play an important role for improving the patient’s personality functioning.

## Conclusion

In this article we illustrated the application of the ICD-11 classification using five different cases in which we took all aspects of the diagnostic guidelines into account. The ICD-11 Personality Disorder classification was found applicable to the five clinical cases, which were classified according to Personaity Disorder severity and trait domain qualifiers. We propose that the classification of severity may help inform clinical prognosis and intensity of treatment, whereas the classification of trait qualifiers may help inform the focus and style of treatment. Empirical research is warranted to investigate such important aspects of clinical utility. Moreover, future empirical research should evaluate perceived ease of use, utility for communication with patients and professionals, and inter-rater reliability. Finally, it seems vital to investigate whether practitioners across all WHO member countries can use the classification in a reliable manner despite substantial diversity in culture and professional resources.

## Additional file

Additional file 1: Essential features and examples of ICD-11 levels of personality disturbance, including “None” and “Personality Difficulty”. (PDF 114 kb)

## Abbreviations

AMPD: Alternative Model of Personality Disorders
DSM: Diagnostic and Statistical Manual of Mental Disorders
ICD: International Classification of Diseases
LPFS: Level of Personality Functioning Scale
LPFS-BF 2.0: Level of Personality Functioning Scale – Brief Form 2.0
PiCD: Personality Inventory for ICD-11
PID-5: Personality Inventory for DSM-5
SCID-AMPD: Structured Clinical Interview for DSM-5 Alternative Model of Personality Disorders

## Footnotes

1 The PID-5 is a 220-item self-report or informant-report inventory developed for the assessment of the trait criterion of the AMPD model (different versions of PID-5 are freely available from psychiatry.org).
