# Supplementary material for: Application of the ICD-11 classification of personality disorders
Source: BMC Psychiatry. 2018 Oct 29;18:351. doi: 10.1186/s12888-018-1908-3 (PMC6206910; doi:10.1186/s12888-018-1908-3)
Supplement: Supplementary file 1 — Essential features and examples of ICD-11 levels of personality disturbance, including “None” and “Personality Difficulty”. (PDF 114 kb) [file 12888_2018_1908_MOESM1_ESM.pdf]

Essential features of ICD-11 levels of personality disturbance

Level	Essential features
None	Stability and coherence of one's sense of identity; ability to maintain an overall positive and stable sense of self-worth; accuracy of one's view of one's characteristics, strengths, limitations. Capacity for self-direction (ability to plan, choose, and implement appropriate goals).<br><br>Interest in engaging in relationships with others; ability to understand and appreciate others' perspectives; ability to develop and maintain close and mutually satisfying relationships; ability to manage conflict in relationships.
Difficulty	Pronounced personality characteristics that may affect treatment or health services but do not rise to the level of severity to merit a diagnosis of Mild Personality Disorder. Personality difficulty is characterized by long-standing difficulties (e.g., at least 2 years).<br><br>Manifested in cognitive and emotional experience and expression only intermittently (e.g., during times of stress) or at low intensity.<br><br>The difficulties are associated with some problems in functioning but these are insufficiently severe to cause notable disruption in social, occupational, and interpersonal relationships and may be limited to specific relationships or situations.
Mild	Disturbances affect some areas of personality functioning but not others (e.g., problems with self-direction in the absence of problems with stability and coherence of identity or self-worth), and may not be apparent in some contexts.<br><br>There are problems in many interpersonal relationships and/or in performance of expected occupational and social roles, but some relationships are maintained and/or some roles carried out.<br><br>Is typically not associated with substantial harm to self or others.<br><br>May be associated with substantial distress or with impairment in personal, family, social, educational, occupational or other important areas of functioning that is either limited to circumscribed areas (e.g., romantic relationships; employment) or present in more areas but milder.
Moderate	Disturbances affect multiple areas of personality functioning (e.g., identity or sense of self, ability to form intimate relationships, ability to control impulses and modulate behaviour). However, some areas of personality functioning may be relatively less affected.<br><br>There are marked problems in most interpersonal relationships and the performance of most expected social and occupational roles are compromised to some degree. Relationships are likely to be characterized by conflict, avoidance, withdrawal, or extreme dependency (e.g., few friendships maintained, persistent conflict in work relationships and consequent occupational problems, romantic relationships characterized by serious disruption or inappropriate submissiveness).<br><br>Is sometimes associated with substantial harm to self or others.<br><br>Is associated with marked impairment in personal, family, social, educational, occupational or other important areas of functioning, although functioning in circumscribed areas may be maintained.
Severe	Disturbances affect most, if not all, areas of personality functioning.<br><br>There are severe disturbances in functioning of the self (e.g., sense of self may be so unstable that individuals reports not having a sense of who they are or so rigid that they refuse to participate in any but an extremely narrow range of situations; self view may be characterized by self-contempt or be grandiose or highly eccentric).<br><br>Problems in interpersonal functioning seriously affect virtually all relationships and the ability and willingness to perform expected social and occupational roles is absent or severely compromised.<br><br>Is often associated with harm to self or others.<br><br>Is associated with severe impairment in all or nearly all areas of life, including personal, family, social, educational, occupational, and other important areas of functioning.

Note. Adapted from the ICD-11 Clinical Descriptions and Diagnostic Guidelines for Personality Disorder. The diagnostic guidelines should be accompanied with the examples on the next page. The threshold for yielding a diagnosis is at least "mild" degree, which therefore may be used as a screener for presence or absence of Personality Disorder.

Examples of ICD-11 levels of personality disturbance

Level	Examples
None	Sense of self is coherent and consistent with how others view them. Manages to recover from injuries to self-esteem and handle different kinds of setbacks in an adaptive manner.<br>Is able to set appropriate goals and to work towards them, to sustain employment and collaborate with supervisors and co-workers, and to understand and appreciate others' perspectives and develop close, mutually satisfying, and long-standing relationships.<br><br>Recognizes, acknowledges, and tolerates a range of unwanted or negative emotions. Flexibility in controlling impulses and behavioral responses.<br><br>Maintains accurate situational and interpersonal appraisals under stress.
Difficulty	In contrast to mild severity, the individual only has some intermittent or low intensity problems (e.g., in circumscribed risk situations), but not in such a way that it compromises the individual's ability to keep a job, initiate and keep friends and have somewhat satisfactory intimate relationships.
Mild	Sense of self may be somewhat contradictory and inconsistent with how others view them; has difficulty recovering from injuries to self-esteem and handling even minor setbacks.; compromized ability to set appropriate goals and to work towards them.<br><br>May have conflicts with supervisors and co-workers, but is generally able to sustain employment; limitations in the ability to understand and appreciate others' perspectives create difficulties in developing close and mutually satisfying relationships; there may be estrangement in some relationships, but relationships are more commonly characterized by intermittent or frequent, minor conflicts that are not so severe that they cause serious and long-standing disruption. Alternatively, relationships may be characterized by dependence and avoidance of conflict by giving in to others, even at some cost to themselves.<br><br>Under stress, there may be some distortions in the individual's situational and interpersonal appraisals but reality testing remains intact.
Moderate	Sense of self may become incoherent in times of crisis; has considerable difficulty maintaining positive self-esteem or, alternatively, has an unrealistically positive self-view that is not modified by evidence to the contrary.<br><br>May exhibit little genuine interest in or efforts toward sustained employment; major limitations in the ability to understand and appreciate others' perspectives hinder developing close and mutually satisfying relationships; problems in those relationships that do exist are common and persistent; may involve frequent, serious, and volatile conflict; and typically are quite one-sided (e.g., very strongly dominant or highly submissive).<br><br>Under stress there are marked distortions in the individual's situational and interpersonal appraisals. There may be mild dissociative states or psychotic-like beliefs or perceptions (e.g., paranoid ideas). Exhibits poor emotion regulation in the face of setbacks, often becoming highly upset and giving up easily. Alternatively, the individual may persist unreasonably in pursuit of goals that have no chance of success.
Severe	Self-view is very unrealistic and typically is highly unstable or internally contradictory; is largely unable to set and pursue realistic goals. Is unwilling or unable to sustain regular work due to lack of interest or effort, poor performance (e.g., failure to complete assignments or perform expected roles, unreliability), interpersonal difficulties, or inappropriate behaviour (e.g., fits of temper, insubordination).<br><br>Interpersonal relationships, if any, lack mutuality; are shallow, extremely one-sided, unstable, and/or highly conflictual, often to the point of violence. Family relationships are absent (despite having living relatives) or marred by significant conflict.<br><br>Under stress, there are extreme distortions in the individual's situational and interpersonal appraisals. There are often dissociative states or psychotic-like beliefs or perceptions (e.g., extreme paranoid reactions). Has extreme difficulty acknowledging unwanted emotions (e.g., does not recognize or acknowledge experiencing anger, sadness, or other emotion). Has serious difficulty with regulation of self-esteem, emotional experience and expression, and impulses, as well as other aspects of behaviour (e.g., perseveration, indecision).

Note. Adapted from the ICD-11 Clinical Descriptions and Diagnostic Guidelines for Personality Disorder. The examples should be accompanied with the diagnostic guideline on the previous page. The specified severity threshold for yielding a Personality Disorder diagnosis is at least "mild" degree, which therefore may be used as a screener for presence or absence of Personality Disorder.
